# Family A and B DNA Polymerases in Cancer: Opportunities for Therapeutic Interventions

**DOI:** 10.3390/biology7010005

**Published:** 2018-01-02

**Authors:** Vinit Shanbhag, Shrikesh Sachdev, Jacqueline A. Flores, Mukund J. Modak, Kamalendra Singh

**Affiliations:** 1 Department of Biochemistry, University of Missouri, Columbia, MO 65211, USA; vcs36d@mail.missouri.edu; 2 The Christopher S. Bond Life Science Center, University of Missouri, Columbia, MO 65211, USA; sachdevs@missouri.edu (S.S.); jaf468@mail.missouri.edu (J.A.F.); 3 Molecular Microbiology and Immunology, University of Missouri, Columbia, MO 65211, USA; 4 Department of Microbiology, Biochemistry and Molecular Genetics 225 Warren Street, NJ 07103, USA; modak@njms.rutgers.edu; 5 Department of Laboratory Medicine, Karolinska Institutet, Stockholm 141 86, Sweden

**Keywords:** DNA polymerase, translesion DNA synthesis, cancer, 3′-5′ exonuclease, replication fork, mismatch repair, base excision repair, therapy for mismatch repair deficient cancers, DNA polymerase and cancer

## Abstract

DNA polymerases are essential for genome replication, DNA repair and translesion DNA synthesis (TLS). Broadly, these enzymes belong to two groups: replicative and non-replicative DNA polymerases. A considerable body of data suggests that both groups of DNA polymerases are associated with cancer. Many mutations in cancer cells are either the result of error-prone DNA synthesis by non-replicative polymerases, or the inability of replicative DNA polymerases to proofread mismatched nucleotides due to mutations in 3′-5′ exonuclease activity. Moreover, non-replicative, TLS-capable DNA polymerases can negatively impact cancer treatment by synthesizing DNA past lesions generated from treatments such as cisplatin, oxaliplatin, etoposide, bleomycin, and radiotherapy. Hence, the inhibition of DNA polymerases in tumor cells has the potential to enhance treatment outcomes. Here, we review the association of DNA polymerases in cancer from the A and B families, which participate in lesion bypass, and conduct gene replication. We also discuss possible therapeutic interventions that could be used to maneuver the role of these enzymes in tumorigenesis.

## 1. Introduction

DNA polymerases conduct DNA synthesis by incorporating deoxynucleoside monophosphate (dNMP) using deoxynucleoside triphosphate (dNTP) as a substrate. These enzymes are indispensable for genome replication, integrity and repair. Because DNA polymerases are necessarily involved in the introduction and amplification of mutations, an understanding of their structure and function is crucial in elucidating the early events of tumor formation. DNA polymerases have been divided into two groups based on their function: (i) replicative and (ii) non-replicative DNA polymerases. Replicative DNA polymerases are required only during cell division to replicate the genome, while the non-replicative DNA polymerases are needed throughout the life-cycle of the cell. An individual cell withstands a daily barrage of endogenous and exogenous DNA modifying agents that cause nearly 70,000 DNA lesions per day [1,2,3]. The ability of cells to tolerate and repair these lesions is dependent on an elaborate DNA repair machinery, which is accomplished in large part through the activity of DNA polymerases.

Since the discovery of the first DNA polymerase (*E. coli* DNA polymerase I) [4,5,6,7,8], many additional DNA polymerases with distinct biochemical properties have been identified. Based upon the conserved sequences, DNA polymerases have been grouped into A, B, C, D, X, Y, RT (reverse transcriptase) and AEP (archaeo-eukaryotic primase superfamily) families [9,10,11,12,13,14,15,16] (Table 1). DNA polymerases that share sequence homology with *E. coli* DNA polymerase I, II, and III have been assigned to the A, B and C families, respectively [9]. DNA polymerases lacking sequence homology with the A, B and C families were grouped into the X-family [9,17]. The D-family polymerases are specific to Archaea [10], whereas Y-family DNA polymerases are found in all kingdoms of life [13]. A separate category of DNA polymerase capable of synthesizing DNA using RNA as a template is the reverse transcriptases (RT) [18,19]. These enzymes are found in retroviruses and humans (human telomerases RT or hTERT).

The Klenow fragment (KF) [20] of *E. coli* DNA polymerase I (pol I) is the prototypical DNA polymerase that has been used to understand the biochemical mechanism of DNA synthesis [21,22,23,24,25,26,27,28,29,30,31,32,33,34,35]. The availability of over expression systems for both KF and pol I [36] facilitated the determination of the first crystal structure of a DNA polymerase [37], and analyses of the kinetic intermediates of DNA polymerase reactions (reviewed in [38]). Importantly, the presence of different conformational states of enzyme/substrate complexes of DNA polymerases seen in the crystal structures [39,40,41] confirmed earlier models based on kinetic studies [38]. The crystal structures of human immunodeficiency virus type I (HIV-1) reverse transcriptase (RT) showed that these structures resembled a half-open, right hand [42,43]. This right-hand configuration has been observed in all DNA polymerases whose structure has been solved. Due to the resemblance with half-open right-hand, individual structural units have been referred to as the thumb, palm and fingers subdomains [42,44,45]. While early structures of DNA polymerases revealed the location of the active site and conserved motifs among DNA polymerases, it was the ternary complex (enzyme/template-primer/nucleoside triphosphate) structure of DNA polymerase β [46,47], which provided insights into the divalent, cation-mediated, nucleotidyltransferase reaction mechanism. Ternary complex structures of T7 DNA polymerase [41] and HIV-1 RT [40] further enhanced our understanding of divalent-mediated nucleotide incorporation.

To date, at least 17 human DNA polymerases have been discovered. These polymerases have been classified into five groups: A, B, X, Y and AEP (archaeo-eukaryotic primase superfamily) [48,49,50,51]. Of these, AEP is the most recently discovered family of DNA polymerases [16]. The AEP family members of polymerases are multitasking enzymes since they can initiate de novo DNA-dependent RNA synthesis, DNA-dependent DNA synthesis, translesion DNA synthesis (TLS), and origin-independent re-priming (reviewed in [51]). TLS is mainly conducted by the Y-family of DNA polymerases. However, recent studies suggest that polymerases belonging to other families can also conduct TLS. In this review, we focus on the A family of TLS polymerases and the B family of DNA polymerases, with an emphasis of their associations in cancer.

## 2. Family A DNA Polymerases

*E.coli* DNA polymerase I typifies A family DNA polymerases. There are three known human DNA polymerases that belong to A Family. These are pol γ, pol θ and pol ν. DNA polymerase γ is the major replicase of mitochondrial DNA, however, another mitochondrion-related DNA polymerase has been recently discovered [51]. Pol γ mutations have been associated with several mitochondrial diseases, and these are reviewed elsewhere [52,53,54,55,56,57].

### 2.1. DNA Polymerase θ

DNA polymerase θ is encoded by the mammalian *POLQ* gene [58,59]. Human DNA polymerase θ consists of 2590 amino acids and has three domains: (i) the N-terminal ATPase/helicase-like domain, (ii) a central region, and (iii) the C-terminal polymerase domain [60]. The C-terminal polymerase domain shares ~30% sequence homology with KF [61] and possesses each of the conserved A, B and C polymerase motifs [59]. The structures of the helicase domain (residues 1–891) and the polymerase domain (residues 1792–2590) have been determined [62,63]. The DNA-dependent ATPase activity of polymerase θ has been shown but the helicase activity is yet to be demonstrated [64]. Polymerase θ also has a 5′-dRP lyase activity that is required for short patch base excision repair (BER) [65]. In regards to TLS, DNA polymerase θ efficiently bypasses apurinic/apyrimidinic (AP) sites by preferentially incorporating adenine (A) opposite to the AP site, and thymine glycols [66]. In vitro studies have shown that polymerase θ can efficiently extend mismatched termini resulting from the error-prone dNMP insertion by Y-family DNA polymerase ι [67], and nucleotides inserted against cyclobutane pyrimidine dimers (CPDs) or pyrimidine–6/4-pyrimidone photoproducts ([6,4]PP) [66]. Knockdown of polymerase θ in mouse CH12 B lymphoma cells has been shown to increase the sensitivity to cross-linking agents (mitomycin C and cisplatin), an alkylating agent (methyl methanesulphonate) as well as UV irradiation [68]. Animal model studies have shown that polymerase θ mutant mice cells were vulnerable to radiation-induced micronuclei formation, but were still viable [69]. The *POLQ*-defective, bone marrow stromal cells were not only sensitive to ionizing radiation and bleomycin, but also showed an increase in micronuclei in red blood cells [70,71]. A comparison of *POLQ* mRNA in tumor tissues and matched control tissues from the same individuals showed higher relative *POLQ* expression in stomach, lung and colon cancers [72]. Furthermore, a study of colorectal cancer patients found that cancer patients with higher expression of a group of 47 DNA-replication-related genes which includes *POLQ* in tumors correlated with poorer patient survival [73]. An analysis on human breast cancers found that of the 14 nuclear DNA polymerase genes, only *POLQ* expression was significantly higher in the cancer tissues in comparison to normal tissues [74]. Another report found highest *POLQ* expression in Estrogen Receptor (ER)-negative and high-grade tumors, with higher *POLQ* levels correlating with shorter relapse-free survival times [75]. POLQ is upregulated in oral squamous cell carcinomas [75], and higher expression of *POLQ* was associated with poor outcome in patients with early to mid-stage non small-cell lung cancers [76]. Similarly, *POLQ* gene expression in ovarian carcinoma shows that its expression correlates with tumor grade [77]. Collectively, these studies have raised a possibility that polymerase θ may be a driver of cancer. Further research will shed light on the precise role of different polymerase θ variants in oncogenesis.

### 2.2. DNA Polymerase ν

The third A family human DNA polymerase is polymerase ν, which is encoded by the *POLN* gene. The C-terminal domain of polymerase ν protein has ~29% sequence homology with human DNA polymerase θ [61], and contains conserved A, B and C motifs [9,11]. A domain with structural homology to the 3′-5′ exonuclease domain of *E.coli* DNA polymerase is also present in polymerase ν; however, the metal-coordinating residues required for proofreading activity are not present in this enzyme. In vitro experiments have shown that polymerase ν is a highly error-prone enzyme [78,79], and bypasses thymine glycol efficiently [79]. Nearly 50% of breast carcinomas have mutations within the *POLN* gene, suggesting that polymerase ν may be associated with breast cancer [80]. A recent report suggests that polymerase ν has some ability to bypass the major groove peptide adducts and residues of the DNA crosslink repair [81]. However, inactivation of *POLN* in mouse embryonic fibroblasts had no effect on cellular sensitivity to mitomycin C, cisplatin, or aldehydes [81]. In human cells, shRNA or siRNA-mediated depletion of *POLN* did not change cellular susceptibility to mitomycin C or alter the frequency of mitomycin C-induced, radial chromosomes [81]. On the surface, these results may suggest a limited involvement of polymerase ν in DNA damage; however, more research is needed to establish the extent to which this polymerase is involved in cancer.

**Figure 1:**
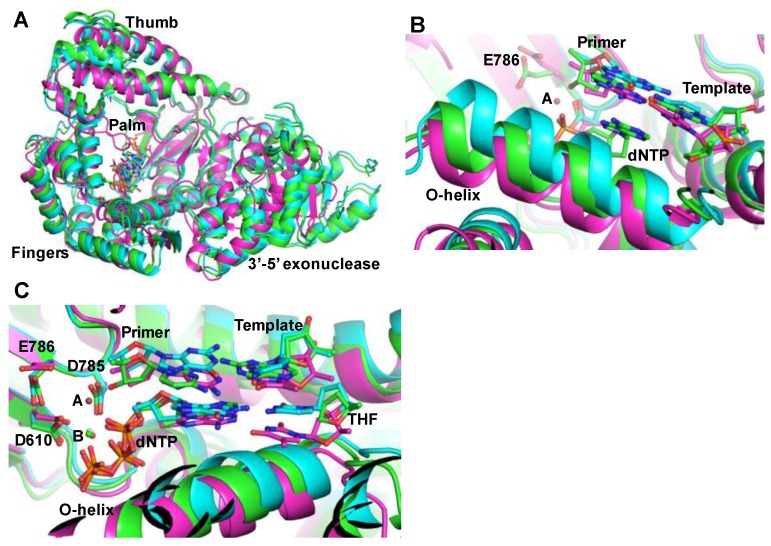
Structures of family A DNA polymerases. (**A**) superposition of the ternary complex crystal structures of polymerase θ [63] (green, tetrahydrofuran-ddATP; cyan, dTMP-ddATP) and the ternary complex of KlenTaq (magenta, Protein Data Bank file 1QSY, Li et al., [83]); (**B**) This figure shows three different conformations of O-helix. Depending upon the template, polymerase θ assumes different O-helix conformation to conduct translesion synthesis; (**C**) close-up of the active site in three crystal structures. Only metal B, which is Ca^2+^ (shown as green ball) was seen in the crystal structures of polymerase θ. Metal A (pink ball) as seen in the crystal structure of KlenTaq is also shown here. The three active site residues of KlenTaq (D610, D785 and E786) are also shown in this figure. For simplicity, the residues positions of only KlenTaq are marked.

Mutations introduced during DNA replication or DNA repair are hallmarks of cancer. Hence, the fidelity of DNA polymerases plays a central role in tumorigenesis. DNA polymerases that have associated 3′-5′ exonuclease activity have the ability to remove errors during DNA synthesis and therefore, have greater fidelity. However, both polymerases (θ and ν) lack 3′-5′ exonuclease activity, which may be a reason for the reduced fidelity of these enzymes. In simplest terms, the fidelity of DNA polymerases is defined as the ratio of polymerase efficiencies [defined as the ratio of catalytic rate (*k_pol_*) and dNTP substrate binding affinity (K_d.dNTP_)] of correct and mismatched nucleotide incorporation. The recently reported crystal structures of the ternary complex (enzyme/DNA/ddNTP) of polymerase θ [63] and the binary complex (enzyme/DNA) of polymerase ν [82] offer insights into the active site conformation, and an explanation for the error-prone DNA synthesis by these two enzymes. In addition, the ternary complex of polymerase θ in complex with a template-primer containing the AP-site analog tetrahydrofuran provides the first glimpse of the TLS by an A Family DNA polymerase [63] (Figure 1). These structures show that the enzymes retain the core architecture of bacterial A family polymerases, but contains additional loops and inserts for specific functions. Comparison of the crystal structures of polymerase θ in complex with template-primer containing tetrahydrofuran (THF) analog and ddNTP with the template-primer and ddNTP showed that the O-helix adopts different conformations depending upon the templating sequence [63]. It is possible that the conformational flexibility of O-helix of DNA polymerase θ permits TLS and error-prone DNA synthesis by this enzyme.

## 3. Family B DNA Polymerases

Human B Family polymerases include α, δ, ε and ζ. The majority of nuclear DNA replication is conducted by α, δ and ε polymerases. These three (α, δ and ε) polymerases function as individual, multi-subunit complexes.

### 3.1 DNA Polymerase α

DNA polymerase α functions as a heterotetrameric complex. The primase active site resides in a p49/p58 complex while the polymerase active site resides in the p180 subunit. Using its primase activity, polymerase α incorporates 7–12 ribonucleoside monophosphates (NMPs) that are extended up to 20–30 nucleotides by the p180 subunit [84]. The polymerase α complex lacks 3′-5′ exonuclease activity, and has moderate fidelity. Mismatched nucleotides incorporated by polymerase α are corrected by the 3′-5′ exonuclease activity of polymerase δ [85,86,87] or the mismatch repair machinery (MMR) [88].

### 3.2 DNA Polymerase δ and ε

Polymerases δ and ε jointly conduct the replication of the entire nuclear genome. Both polymerases function as holoenzymes composed of multiple subunits. Mammalian polymerase δ is a heterotetramer. The large subunit (p125), which is encoded by the *POLD1* gene, harbors both the polymerase and the 3′-5′ exonuclease domain. The other three subunits, namely p50, p68 (also called p66), and p12 are regulatory proteins, and they are encoded by *POLD2, POLD3* and *POLD4*, respectively [89]. DNA polymerase δ is responsible for synthesizing the lagging strand [90]. Recent reports suggest that polymerase δ may also be involved in replicating the leading strand [91]. Human polymerase ε also exists as a heterotetramer. The large catalytic subunit (p261), which is encoded by the *POLE1* gene, contains both the polymerase and the 3′-5′ exonuclease activity. The other three subunits are encoded by *POLE2, POLE3* and *POLE4* genes. These subunits act as regulatory proteins or bind dsDNA [89]. Polymerase ε is known to replicate the leading strand of the replication fork [88,92].

Both polymerases δ and ε carry out high fidelity DNA synthesis, which, in part, is facilitated, by their 3′-5′ exonuclease activity. It has been shown that the 3′-5′ exonuclease activities of the two enzymes can function in trans [93], and any mutation affecting proof-reading activity can lead to genome instability. In line with this notion, the polymerase mutator alleles have been shown to increase the risk of human cancer [94].

Several mutations within the DNA polymerase δ catalytic subunit have been reported in colorectal cancer [95], colon cancer [96] and in a rat hepatoma cell line [97]. Mutation D502A was found in the normal colon of a patient who did not develop colon cancer until the age of 70 [95]. Another mutation R506H has been found in the colorectal cancer cell lines DLD-1/HCT15 [95]. The polymerase domain mutation R648Q was detected in rat hepatoma cells [97]. Of the six conserved exonuclease motifs (Exo I to Exo VI) that have been identified [98,99,100], amino acid residues 502 and 506 belong to the Exo III motif of polymerase δ, whereas R648 is located in the ‘fingers’ subdomain. The activity of partially purified human polymerase δ containing R648Q mutation reduced the fidelity of DNA synthesis [101,102], suggesting a role of R648 in the recognition of correct nucleotide. In addition, a frameshift mutation in the HCT116 colorectal cancer cell line has been identified [96]. This polymerase δ variant that lacks two conserved carboxy terminus DNA binding domains. This polymerase δ variant is also expressed at decreased levels in mutant cells [96]. As depletion of polymerase δ is known to affect chromosomal instability in yeast [103,104], it is likely that mutations in polymerase δ that reduce fidelity or its abundance may contribute to DNA changes that accompany tumorigenesis.

As with polymerase δ, mutations in the 3′-5′ exonuclease domain of polymerase ε has also been identified in different tumors [105,106,107]. Sequencing of genomic DNA encoding the 3′-5′ exonuclease domain of *POLE* from a set of 76 colorectal carcinomas and six colorectal cell lines identified F376S mutation in one patient [108]. F367 is a conserved residue within the Exo II domain in family B DNA polymerases. Topologically, the F367 equivalent residue in bacteriophage RB69 replicase is located adjacent to the metal B coordinating carboxylate (D222) [109]. A study reporting comprehensive molecular characterization of human colon and rectal cancers by the Cancer Genome Atlas Network showed that about 4% of tumors classified as microsatellite stable had mutations in the exonuclease domain of *POLE* [110,111]. Several other studies have reported mutations in polymerase ε in different types of tumors including colorectal adenomas and carcinomas [112,113,114,115]. Screening of the 3′-5′ exonuclease domains of *POLE* (residues 268–471) in 173 endometrial cancers resulted in the identification of 13 non-synonymous variants in *POLE* [116]. In addition, the percentage of endometrial cancers with polymerase ε somatic mutations was around 8% [116]. Of the 13 mutations, somatic mutations D275V and V411L and germline mutation R311C are specifically interesting. D275 is an active site aspartate of 3′-5′ exonuclease activity and coordinates with metal A, whereas V411 is at the DNA binding interface. Germline mutation R311C is in the vicinity of the Exo I conserved motif.

Numerous studies have demonstrated that DNA replication fidelity is critical in cancer susceptibility and development. Inhibition of the proofreading activity of DNA polymerase δ contributes to a distinctive spectrum of cancers as compared to inhibiting DNA polymerase ε. Polymerase δ and ε proofreading deficient mice showed different survival rates and surprisingly tissue-specific tumor susceptibility [117]. Mice with a *POLD1* mutation exhibited thymic lymphomas and skin sarcomas, whereas mice with *POLE* mutation had histiocytic sarcomas and nodal lymphomas [117]. In one of the earlier studies by Flohr et al. [96] several colorectal cancer cell lines (DLD-1, HCT 116, SW 620, SW 480, SW 48, HT 29) and samples from colorectal cancer patients were screened for a mutation in DNA polymerase δ. Most of these cell lines and patient samples had a mutation in the mRNA of DNA polymerase δ that was expected to modify the structure of the enzyme and causing a defect in the proofreading activity potentially contributing to a high mutation rate commonly observed in colorectal cancers [96].

*POLD1* and *POLE* are the largest domains of polymerase δ and polymerase ε respectively, in humans that have catalytic proofreading exonuclease activity. Recently, many exonuclease domain mutations (EDMs) in human *POLD1* and *POLE* have been identified that directly correlate and predispose to “polymerase proofreading associated polyposis” (PPAP), a disease characterized by multiple carcinomas [106]. Some of the mutations are enlisted in Table 2. The structure of *POLD1* and the location of residues that are mutated along with evidence in pathogenicity, molecular characteristics and occurrence in tumor types have been nicely reviewed recently [118]. Over the past 5 years, studies have shown that germline mutations in these domains predispose to colorectal cancers and other malignancies [108]. Cancer genomes of children′s that acquired biallelic mismatch repair deficiency (bMMRD) exhibited massive amounts of mutations than all childhood and most cancer genomes that were analyzed in the study. All the bMMRD cancers had a somatic driver mutation in either polymerase δ or polymerase ε [119]. The somatic and germline mutations in humans and their correlation to cancer, especially colorectal and endometrium cancer, is a recent finding and is very intriguing [113]. However, more work needs to be done to understand the molecular basis on how these mutations enhance the process of tumorigenesis with an endgoal of developing novel treatment strategies. Additionally, sequence analysis for similar mutations in other forms of cancer and classification based on their origin (spontaneous or hereditary) will allow for better genetic testing and clinical surveillance ultimately leading to better clinical outcomes.

### 3.3. Polymerase ζ

Human DNA polymerase ζ was initially discovered as a dimeric enzyme consisting of catalytic subunit Rev3 and the structural subunit Rev7 [120,121,122]. Subsequently, a four-subunit polymerase ζ complex containing Rev3, Rev7, p50 and p66 was discovered [123,124,125]. Subunits p50 and p66 are also part of the polymerase δ holoenzyme. An interaction between the polymerase ζ structural subunit Rev7 and Rev1 has also been documented [126], and this interaction appears to be functionally important for TLS across a (6–4) TT photoproduct [127]. Polymerase ζ is a low fidelity polymerase, and does not possess 3′-5′ exonuclease activity. Initially polymerase ζ was considered a TLS polymerase, a concept that was based on the observation that the yeast polymerase ζ was able to perform DNA synthesis past a *cis-cyn* TT dimer [120]. However, later studies showed that polymerase ζ itself was unable to synthesize DNA past a lesion [49,122]. It is now believed that polymerase ζ is an “extender polymerase”, which extends the lesion bypassed by Y-family polymerases such as η, ι or κ [49].

The relevance of polymerase ζ in cancer is associated with its role in TLS. It has been shown that polymerase ζ is involved in bypassing cisplatin-GG [128], OBPDE-GG [128], (6,4) TT photoproduct [128,129,130], AP sites [128] and thymine glycol [131]. Polymerase ζ has also been demonstrated as a major determinant for resistance to platinum based anti-cancer compounds [132]. Both in vivo and in vitro studies have demonstrated that decreased *REV3* expression increased the sensitivity of lymphoma to cisplatin [133]. Knockout of *REV3*, the gene encoding the catalytic subunit of polymerase ζ in mouse embryonic fibroblasts, increased chromosomal instability in a p53-dependent manner [134]. Loss of *REV3* enhanced spontaneous tumorigenesis in a p53-deficient background [135]. Interestingly, overexpression of *REV3* may have the same effect since it was found to increase breast cancer tumor cell migration and invasion. Singh et al hypothesized that *REV3* could be protecting the tumors from DNA damage or creating mutations through TLS [136]. A breast cancer epidemiology study found that *REV 1* and *REV 3* single nucleotide polymorphisms (SNPs) not only affected the kinds of tumors that Swedish patients had but also their chances of surviving. Specifically, minor alleles of the *REV1* SNPs rs3792142 and rs6761390 were associated with larger tumors and advanced stage cancer. Meanwhile, the CC variants of the *REV 3* SNPs rs11153292 and rs462779 reduced patients’ odds of surviving [137].

## 4. Potential Therapeutic Interventions

Accumulation of a higher number of mutations in cancer can usually stimulate the production of “non-self” antigens [138]. This mechanism of producing non-self antigens makes the tumor vulnerable to attack by immune cells. However, cancer cells acquire escape machinery by expressing protein molecules that allow them to remain undetected by the immune surveillance system [138]. The expression of immune checkpoint protein molecules, Programmed Death 1 (PD1) on T regulatory immune cells and PD-L1 and PD-L2 on normal and cancer cells allows the cells to pass the checkpoint and survive. Thus, immune checkpoint blockade has offered remarkable success in treating many forms of cancer [139,140,141,142,143].

A recent study assessed the expression of PD-L1 expression in mismatch repair deficient endometrial tumors and showed a significant increase in its expression than other mismatch repair proficient counterparts [144]. Importantly, blocking the immune checkpoint has recently been shown to be a promising treatment in colorectal cancer patients with mismatch repair deficiency (dMMR). In this study, pembrolizumab an anti-PD1 inhibitor significantly benefitted the cancer patients with mismatch repair deficiency than the patients with tumors that have mismatch repair proficiency [145]. Another recent phase II trials- Checkmate 142 assessed the use of a well-tolerated Nivolumab anti-PD1 inhibitor in patients with metastatic colorectal cancers and mismatch repair deficiency [146]. Considering the stark similarities between the cancers with (dMMR) and the ones with a defect in proofreading exonuclease activity [147,148,149], the immune checkpoint blockade offers a unique opportunity in the treatment of cancers that have mutations in polymerases lacking proofreading activity. However, more work needs to be done to identify predictive biomarkers that would select patients for such kind of immunotherapy and spare nonresponders from potential side-effects. Also, the novel antigens that are produced by large accumulations of mutations in tumors caused by mismatch repair deficiency is an obvious weakness that can be exploited using a combination of exome based identification of novel antigens and selectively enhancing the activity of cytotoxic T cells against these antigens in tumors [150].

Recently, it was demonstrated that DNA polymerases δ and ε could be a potential candidate as targets for gene therapy in hepatocellular carcinoma (HCC). In this study, microRNAs targeting DNA polymerases δ and ε were used to block the proliferation of HCC cells using a controlled tumor-specific promoter system. This approach might be used to block the function of mutated/dysregulated polymerases specifically in cancer cells potentially avoiding the off-target specificity [151]. Another strategy that could be employed to treat cancers with polymerase proofreading deficiency is to increase the number of mutations in tumors to a level that exceeds a threshold of the cancer cells not allowing them to grow. In support of this concept, cadmium has been shown to increase the lethality of yeast that expresses a proofreading deficient polymerase δ with no effect on WT strains [152]. It has also been demonstrated that altering the dNTP pools significantly affects the sensitivity of yeast strains that express the Pol δ and Pol ε exonuclease domain mutations [153,154]. Hence, in tumor types with mild mutations, inhibiting the dNTP pools might result in a reduction in tumor adaptation. Conversely, dNTP pools can be increased to levels that exceed the mutation threshold in tumors with a higher number of mutations. However, DNA damage response could be different in yeast and humans and hence more work is warranted to test certain mutagenic compounds that might exacerbate the lethality of cancer cells leading to better clinical outcomes.

Many mutations that cause a defect in the exonuclease activity of polymerases δ and ε have been identified in in vitro and in vivo models. With the advent of CRISPR Cas-9 gene editing system [155] gain of function mutation could be achieved. It is too early to predict the clinical outcomes using this technology, however, this approach could also be used in near future to regain the proofreading activity of polymerases that have a crucial role in suppressing the activation of tumorigenesis. Targeting the polymerase using small molecule inhibitors to suppress its function can be an additional therapeutic intervention in cancer.

## 5. Concluding Remarks

There is growing appreciation for the various ways in which DNA polymerases play a role in genome instability. In the case of non-replicative DNA polymerases such as polymerase θ, small molecule inhibitors can be developed to specifically target these enzymes in tumor cells. However, different strategies like gene therapy or gene editing would be required to correct subunit specific SNPs in order to restrict tumor growth. For tumors associated with mutations in replicative polymerases immunotherapy approaches could be utilized for better recognition of tumor cells by the host immune systems. Additionally, CRISPR Cas-9 gene editing system can be applied to restore normal 3′-5′ exonuclease function. Further in vitro and in vivo characterization of known and newly discovered DNA polymerases will provide additional insights into their function in normal and tumor cells, which can lead to new therapeutic interventions against variety of cancers.

## Figures and Tables

**Table 1:** Polymerase families and representative DNA polymerases.

Family	Prokaryotic ^a^	Eukaryotic	Archaea	Virus
A	Pol I	Pol γ, θ, ν		T3, T5, T7 pol
B	Pol II	Pol α, δ, ε, ζ	Pol BI, BII	RB69, T4 pol
C	Pol III			
D			Pol D	
X		Pol β, λ, μ		
Y	Pol IV, V	Pol η, ι, κ		
RT	hTERT	Telomerase		Reverse Transcriptase
AEP		Prim-pol		poxviruses, asfarviruses, iridoviruses, phycodnaviruses mimivirus

^a^ hTERT, human telomerase reverse transcriptase; RT, Reverse Transcriptase; AEP, Archaeo-Eukaryotic Primase.

**Table 2:** Mutations in 3′-5′ exonuclease domain of polymerase δ and ε and their predisposition to the cancer type.

Polymerase δ	Predisposition to the Cancer Type	Polymerase ε	Predisposition to the Cancer Type
C319Y	Multiple myeloma and Glioblastoma	D275V	Endometrial
D316G	Colorectal, endometrial and breast	E277	Endometrial
D316H	Colorectal, breast, and mesothelioma	P286R/H/S	Colorectal
L474P	Colorectal and endometrial	S297F	Ovarian
R409W	Colorectal	F367S in	Colorectal
S478N	Colorectal and endometrial	V411L	Colorectal
P327L	Multiple adenomas	L424V	Colorectal
		P436R/S	Colorectal
		M444K	Colorectal
		A456P	Colorectal
		S459F	Colorectal
